# Pain management after third molar extractions in adolescents: a qualitative study

**DOI:** 10.1186/s12887-022-03261-x

**Published:** 2022-04-07

**Authors:** Shannon Gwin Mitchell, Anjali R. Truitt, Lauryn M. Davin, D. Brad Rindal

**Affiliations:** 1 Friends Research Institute, Inc, 1040 Park Avenue, Suite 103, Baltimore, MD 21201 USA; 2 HealthPartners Institute, 8170 33rd Ave S, Bloomington, MN 55425 USA

**Keywords:** Analgesics, Adolescent, Third molar extractions, Pain, Postoperative, Dentistry, Prescriptions, Health communication, Oral, Decision-making

## Abstract

**Background:**

Understanding how adolescent patients make decisions about pain management after complex dental procedures could help reduce the use of opioid medications and the potential for future opioid misuse in this population. This qualitative study explores how adolescents manage pain, including how decision making with parents and providers affect their experience with opioid and non-opioid analgesics after third molar dental extractions.

**Methods:**

We used a qualitative approach for the analysis of 30 telephone-based semi-structured interviews completed by 15 dyads between May and August 2019, which included 15 adolescents (15–17 years) who underwent a dental extraction, and a parent or guardian for each adolescent. The total sample included 30 participants. Interviews were conducted separately for patients and parents. De-identified interview transcripts were analyzed using qualitative analysis software using a directed content analysis approach.

**Results:**

A total of 15 patient/parent dyads were interviewed. Key themes associated with pain management included sources of information, pain management behaviors engaged in by the adolescents and their caregivers, and the use of medication. In addition to conversations with their dental provider, most patients and their parents discussed pain management plans that included non-medication options, over-the-counter medications, and opioid medications to be taken as needed, which guided their post-extraction behaviors. All participants reported that the adolescent received an opioid prescription for post-extraction pain management, to be taken on an “as needed” basis, but most only took it the day of the extraction and up to 2 days following, usually based on the patient’s reported pain levels and perceptions of over-the-counter medication adequacy. Participants said they did not receive guidance from their provider concerning disposal of unused opioid medications.

**Conclusions:**

Involving adolescents in the pain management decision making process and allowing them to carry out the plan with some caregiver support was acceptable and well executed following third molar extractions. Providers may have an opportunity to reduce the number of opioids prescribed, since respondents reported little to no use of opioids that they were prescribed. Providers should educate patients and their parents about safe disposal of opioids to mitigate the potential for diversion.

**Supplementary Information:**

The online version contains supplementary material available at 10.1186/s12887-022-03261-x.

## Background

Opioids are currently the most commonly prescribed class of medications for the treatment of acute as well as chronic pain in the United States. However, according to the Centers for Disease Control and Prevention, these medications make a significant contribution to our nation’s epidemic of fatal and non-fatal overdoses [1–4]. Exposure to an opioid before completing high school graduation is independently associated with future opioid misuse among low-risk children, making pain management an important topic for examination among pediatric populations, such as adolescents [5].

Such findings are particularly relevant to dentistry, in that an estimated 5 million people undergo third-molar extractions in the United States each year [6]. The majority of dental practitioners report prescribing opioid medications, predominately hydrocodone, following third molar extractions [7]. Because third molar surgery is more difficult as patients age, the American Association of Oral and Maxillofacial Surgery recommends removing third molars associated with disease, or at high risk of developing disease, by a patient’s mid-twenties [8]. Dentist–prescribed opioids account for nearly one-third of the opioid prescriptions for 11–19 year olds [9], making dentists the highest opioid prescriber by specialty for this age group [10]. While the overall rate of dentist-prescribed opioids has decreased across all age groups [11], from 2010–2015, the number of dentist-prescribed opioids for 11–18 year olds increased [12].

Clinical practice guidelines for all patients, including adolescents, aim to reduce opioid prescribing (and the possibility of misuse or abuse of such substances by patients) while adequately and appropriately managing perioperative pain after third molar extractions. Such guidelines focus specifically on increasing providers’ awareness of optimal opioid prescribing [10, 11]. Although factors that influence adherence to pharmacological pain management in older adults has been investigated [13], little is known about how younger patients’ knowledge, attitudes, and beliefs influence decision-making about opioid use following third molar extractions. Questions regarding the patient influence on opioid prescribing (e.g., are they expecting to be in pain and receive opioids for treatment, how many tabs and for how many days to prescribe them) are particularly complex when the patients are adolescents.

Unlike adult patients, who share decision-making with their provider alone [14, 15], adolescent patients often make healthcare decisions in conjunction with their parent or guardian. In the interplay between patients, their caregiver, and dentists, each may impact knowledge, attitudes and beliefs shaping opioid use following a third molar extraction. Improving decision-making about perioperative pain management may reduce opioid prescribing (by dentists), opioid use (by patients) and, when involving minors, increase adherence to the agreed-upon analgesic plan (based on patient buy-in relative to other options and parental support and encouragement) and satisfaction with care [16].

Given the higher risk for substance use among adolescents [5] and the opportunity to decrease expectancies regarding the need for opioids by patients or their caregivers, as well as increased knowledge of the risks associated opioid use, this qualitative study explores how adolescents manage pain, including how decision making with parents and providers affect their experience with opioid and non-opioid analgesics after third molar extractions. Better understanding regarding patients’ and their families’ experience with perioperative pain associated with third molar extractions may improve how dentists communicate family-centered strategies to reduce opioid prescribing, use, and potential diversion.

## Methods

This study was conducted in HealthPartners, a nonprofit health care system in Minnesota, and was approved by HealthPartners Institute Institutional Review Board. The procedures followed were in accordance with the ethical standards of the responsible committee on human experimentation. Informed verbal consent was obtained from all adult participants for their own and their child’s participation, and informed verbal assent was obtained from all participants under the age of 18, which covered all study-related activities.

Recruitment and consent data were collected and managed using REDCap electronic data capture tools hosted at HealthPartners Institute. REDCap (Research Electronic Data Capture) is a secure, web-based software platform designed to support data capture [17]. For this project, it provided an intuitive interface for validated data capture across multiple roles and users.

### Eligibility

Potential participants were identified based on age (years of age 15–17) and recent (< 8 days) permanent tooth extraction procedure using a data query of the HealthPartners electronic health record. Any patient who opted out of research participation or did not have a phone number on record was not considered eligible.

### Recruitment

Patients’ parents or guardians received an invitation letter inviting them to participate in the project and describing the study procedures, offering the opportunity to opt-out, and informing the household that they would receive a call from the study team. Households were called a week after mailing the letter. Up to one call a day was made at different times of day, and days of the week, in order to maximize the potential of reaching eligible participants within the interview window (up to 30 days post-extraction). A voicemail was left on the first and last call attempt. Parents or guardians who agreed to participate, where informed of the study and immediately were verbally consented over the phone. After an adult consented, the study team member would invite the adolescent to participate and obtained verbal assent. Interviews could be done immediately after consent/assent or the research team member could schedule a different time for the interview. After interview completion, each respondent received a $50 gift card to thank them for their time.

### Data collection

As planned, a total of 30 participants were interviewed (15 adolescent patients and a caregiver/parent for each patient) between May and August 2019. A trained qualitative interviewer conducted all interviews telephonically, with interviews averaging approximately 15 min in length. When possible, the interviews were performed separately, so that respondents felt free to respond differently than their pair. Semi-structured interview guides were created for the adolescent patients (Appendix A) and for the caregivers (Appendix B). Each interview guide included core questions asked of all respondents with suggested “probes” to elicit greater details, when necessary. The experienced, trained interviewer consulted the interview guides throughout the interviews to ensure consistency of topics addressed. The interview guide was developed to address the following content areas: knowledge, attitudes, and expectations concerning the dental procedure; pain management decision-making; pain management behaviors post-extraction, and exploration of how these factors influenced treatment. All interviews were audio-recorded, with express permission from the participants, and transcribed verbatim.

### Data analysis

De-identified transcripts were loaded into Atlas.ti 8 [18], a computer assistive qualitative analysis software, where a directed content analysis was applied [19]. Using a constant comparative approach [19], three independent analysts (the first three authors of this paper) reviewed emergent themes and repeatedly revisited the data to detect outliers and exceptions. (See Appendix C for the study’s Code Book with a list of codes and definitions). During the initial phase, they performed open coding where they segmented the data into similar groupings and formed preliminary categories regarding pain experiences, pain management, and share decision making, largely embedded in the interview guide questions. After coding the first transcript the three analysts met to compare codes, develop code definitions, and resolve any discrepancies. This process was repeated with two additional transcripts and then, once the codes and coding procedures were well established, one team member completed coding on the remainder of the dataset. During the second phase, the team of 3 analysts similarly performed axial coding, where they began to assemble categories, building logical connections or relationships among codes to develop more detailed thematic construction. As with the initial phase, a total of three transcripts were comparatively coded and discussed until consensus had been reached. During the final phase, the team performed selective coding where they clarified categories and themes and organized the themes to articulate a theory regarding the phenomenon of interest. The coders communicated regularly about the codes and their applications. The full analytic process took approximately 3 months to complete.

## Results

Thirty-eight households were mailed a notification letter, of which one had an inaccurate mailing address. Thirty-seven households entered phone follow-up. Of these, ten were unreachable and four guardians declined participating, five were ineligible (e.g., patient turned 18 years old, parent was dental care provider at HealthPartners). Eighteen guardians consented, but in three instances, the patient was unavailable or unreachable within the maximum number of call attempts or within 30-days post extraction. Fifteen dyads completed an interview.

The patient demographics are summarized in Table 1. The patient sample was 53% female; 53% were receiving Medicaid/subsidized state health insurance; about half were 16-years of age with one 15-year-old and the rest 17-years of age; 53% were White, with one identifying as Asian, and 40% identifying as more than one race, and two identifying as Hispanic/Latino. Ethnic/racial demographics were not collected for caregivers.

**Table 1:** Patient demographics (*n* = 15)

Characteristic	% (n)
**Female**	53 (8)
**Age**	
15	7 (1)
16	53 (8)
17	40 (6)
**Race**	
White	53 (8)
Asian	7 (1)
More than one race/other	40 (6)
** Hispanic/Latino**	13 (2)
** Medicaid/state subsidy**	53 (8)

Key themes associated with pain management included sources of information, pain management behaviors engaged in by the adolescents and their caregivers, and the use of medication.

### Sources of information

Decision making involves gathering and assessing information, and adolescents identified parents and other family members, their dental providers, and their peers as sources of information for what to expect in terms of pain intensity and pain management during and after the dental procedure, with parents reporting similar sources of information. Adolescents who received information from peers described it as more general information regarding the procedure and recovery (e.g., “their cheeks kind of swollen after the extraction”, “be careful and cautious about eating”). Parents and other family members, including older siblings, were identified as sources providing more detailed information, sometimes with a cautionary tale. As was the case with peers, this information was usually based on a parent’s/older siblings’ own experiences, having gone through the same procedure or another surgery-related experience requiring sedation.

“Interviewer: Do you want to tell me a little but about your experience? Parent: It was awful. They had a hard time waking me up, and then when I did wake up, every time I got up, I felt like I was going to vomit. They finally had to wheel me out I don't know how many hours later. One of the reasons why I had the conversation with [Child’s name] about really thinking twice about having general for a tooth extraction.”

Dentists and oral surgeons provided information on both the procedure and what to do post-extraction to aid in recovery, and they shared this information with parents and adolescents both before the procedure and again right after the procedure, verbally and through handouts and post-operative information sheets.

“Interviewer: How did the dentist or oral surgeon advise you about managing your child's pain after the surgery? Parent: They told me about the dry socket. They told me to tell him, showed me how to rinse his mouth and about what to eat, gave some suggestions about pain meds like alternating Ibuprofen and Tylenol-Codeine, so they gave some good, a couple, some basic good advice.”

While the information shared by peers was taken as a way to minimize or generalize the dental extraction experiences, caregivers often warned the adolescents of negative outcomes. Information shared by dental providers was highly tactical and was not described as conveying the emotional resonance of the caregivers’ information. These sources of information may have helped prepare the adolescent patient for the pain they were likely to experience from the oral procedure and how to manage it during their post-operative recovery.

### Pain management behaviors by adolescents and their caregivers

Adolescents and parents alike largely described collaboratively making pain management decisions, where they discussed pain management options together and a plan was reached that was agreeable to both.

“Interviewer: So, you decided not use any of the [opioid] medication. Did you talk about that with your parents, or did you just decide yourself? Patient: I was talking about it with my parents.”

Parents largely assisted with organizing the placement and the timing of taking the medications or reminding the child to “stay ahead” of the pain after a plan had been decided upon.

“Parent: I will say that with my experience, I talked to her about it. She can handle it. And also, about the pain but being a mum, I have to monitor the time for the medicine because even she's a little bit older. Sometimes she just forgets.”

In one extreme example a parent (with a health care background) encouraged their child to take an opioid medication, instructing the child to set an alarm to self-medicate in the middle of the night rather than waking up in pain and losing the rest necessary to recuperate. This parent provided a high degree of guidance and control over the pain management process; whereas, the other parents largely gave reminders and support but let their child decide when or if they needed more pain medication and what kind to take.

“Patient: Yeah, if it did end up hurting a lot and I would need it, I would just ask for it and they would bring it.”

Two-thirds of the adolescents (*n* = 10) administered their own non-opioid medication. Adolescents and parents explained that this was largely the result of logistical issues (e.g., the parent or adolescent being at work and unable to rely on one another) and the adolescent being responsible enough to handle the dosing. Parents often provided reminders and other structure in the days following the extraction, such as putting the medications on their bedside.

“Patient: Like for the couple two days, my mom would help me and give me the pills. And then the rest of the days, I would just know when to take it. I would take it myself.”

Eighty percent of parents (*n* = 12) reported keeping the opioid prescription secured. By doing this, they were able to control when and how it was administered. Input from the adolescent about pain intensity was the most common driver for when opioid medications were given.

The main reason for allowing adolescents to self-medicate was that only they could determine how bad the pain was and whether they needed another dose of pain medication. The following quotes (from un-matched parent and child interviews) illustrate this point.

“Interviewer: How would you say you felt about how much input your child had regarding managing their pain? Parent: I think she had enough input. She was really the driver of it. I mean, whatever she needed, we supported her on what she needed for it.”

“Interviewer: And did you remember discussing with your parents whether or not to take those [opioid] medications? Adolescent: Yeah. Interviewer: What went on there? In that conversation? Adolescent: That if I needed them [opioid medications], if I needed to take it because of the pain, I could take them. But I never really went through that much pain, so I just never took them.”

Parents largely deferred to their child when it came to pain management behaviors and, despite not wanting them to be unnecessarily in pain, parents left it up to their adolescents’ discretion.

“Parent: Not much, actually. He was pretty responsible. I mean, that's the one thing is he ... I told him, ‘Hey, if you don't take it, you're going to be in pain.’ Being a 16-year-old, he needs to ... to me, I think he needs to learn how to, because I don't want to tell him to take his pill if he doesn't need to.”

### The use of medications

All participants reported receiving guidance from the dental provider regarding the use of medications for pain management, in addition to receiving antibiotics to assist with the post-operative recovery process. Participants were amendable to following the dental providers’ guidance, as pain medication recommendations usually indicated that they be taken on as “as needed” basis with dosing limitations based on medication dosing guidelines. Recommended medications included both over-the-counter products (e.g., ibuprofen, acetaminophen) and prescription opioids (e.g., Vicodin, Tylenol with codeine), but few participants could specifically name the opioid they were given. As one adolescent recalled,

“Patient: And there was one [medication], but it was for in case I had major pain. It was an opioid. Yeah, I forgot what it was called, but it was in case I had some major pain going on.”

This quote illustrates how the provider gave the patient analgesic options and allowed the patient to decide whether their pain level was severe enough to warrant taking an opioid medication. Either the parent, adolescent, or both from each dyad reported being given an opioid for pain management from their dental provider. While no one reported not filling their opioid prescription, many reported not taking them at all or only taking a couple pills in the first few days following the extraction. Since the prescriptions were to take the medication “as needed” they were taken as prescribed. One parent described the instructions as follows:

“Interviewer: And do you remember how the dentist or oral surgeon sort of advised you about how to manage the pain after surgery? Parent: Yeah, just like Ibuprofen ... you know ice, and [they] gave him a couple of ... again I don't know if it Percocet, Vicodin- Hydrocodone. [They] gave him four. I think of the four he only ended up taking two. One each night, for two nights, just so he could sleep. Interviewer: Mm-hmm (affirmative). Parent: But otherwise, he managed the pain without anything else and within- within a week, well, maybe five days, not even, he was feeling pretty good.”

Another parent described both the medications and timing similarly:

“Interviewer: How did the dentist or oral surgeon advise you or your child about managing the pain after the surgery? Parent: Well, [they] just said just use Tylenol, especially the first few days or whatever because her [inaudible], and then [they] also gave out some heavier dose of pain medicine, and [they] says, ‘Well, you might want to save these until a few days later when the gas wears off and stuff and actually she's feeling the pain,’ which she didn't need to use at all, so that was a good thing, but yeah, they explained both medications to me.”

Despite all participants indicating they had been given opioids to take for pain management as needed, not all participants were aware of being given clear instructions about disposal of unused medications. Some participants were given guidance by pharmacists upon filling the prescription, while others were not. Some participants asked about disposal on their own. No participants reported receiving direction on disposal of unused medications from their dentist or oral surgeon.

## Discussion

While adolescents receive information regarding dental surgery from multiple sources, including their friends, their dental provider, and their parents and older siblings, it is their parent/caregiver that they ultimately turn to for informational support and structure in the post-operative period. Reaching a mutually acceptable plan concerning pain management means the parents have input, but ultimately the adolescents in our sample were responsible for managing their pain and following post-operative instructions. While this was often done for practical reasons (e.g., the parent not being with the child continuously), since pain is a subjective experience this reliance on the adolescent’s perceptions of pain to guide dosing is both understandable and appropriate [20, 21]. However, parents in our sample sometimes anticipated their child’s pain level based on their own personal experiences with the same or other surgical procedures. Since pain expectations have been found to be associated with both perceived pain and patient satisfaction levels [22], dental providers may want to explain to adolescents and their parents that differences in pain sensitivity impact post-operative pain. Expectations of post-operative pain should emphasize typical responses rather than the extremes, and adolescents should be encouraged to share their perceived pain levels with their parents/caregivers to help guide and improve their pain management.

The adolescents’ pain management behaviors followed the guidance of their dental providers, which allowed for patient input concerning which analgesic medications to use and the duration of use. Adherence to dental treatment recommendations has been shown to be associated with reduced complications in other pediatric and adolescent samples [23]. Parents provided reminders and some support immediately following the procedure but the adolescent patients were given some autonomy in the process. While participants reported the adolescent receiving some form of opioid medication following surgery and filling the prescription, few reported taking all, and some did not take any, of the opioid medication. Our findings also support those of Maughan and colleagues [24], who found that over half of the opioids prescribed following a dental procedure went unused. While their study identified that an average of 28 opioid pills were prescribed to adult participants by their dental providers at the time the study was conducted, and that average opioid prescriptions may be lower now, given the American Dental Association’s 2018 opioid prescribing policy [25], our sample still noted that they were left with unused opioid medications in their home since the OTC medications and other treatments (e.g., icing the jaw to reduce inflammation) were sufficient for managing post-operative pain. Rather than needing 7 days of opioid pain medications, our sample reported using them no more than 2 or 3 days, at most, when there were no other post-operative complications.

### Improving clinical practice

Since pain following a dental extraction is primarily due to inflammation and nonsteroidal anti-inflammatory drugs target inflammatory pathways, they are generally effective at managing pain following a dental extraction. Clinical trials show that they are at least as effective as opioids. The evidence would suggest that opioids should only be prescribed for the managing only the most severe pain [26, 27]. Our findings indicate that dentists may have opportunities to discuss increasing the dose of nonsteroidal anti-inflammatory drugs and combining them with acetaminophen if the pain is not controlled. Rather than providing patients with any opioids in case of poor pain control, a discussion regarding dosing adjustments to the non-opioid analgesics and adding this information to the instructions sent with the patient should be the norm in clinical practice. Opioid analgesics should be considered only when nonsteroidal anti-inflammatory drugs or acetaminophen are not safe due to known side effects, medical conditions, or drug interactions. If an opioid is prescribed, information should be communicated and sent with the patient instructing them on the disposal of unused medication. Given the autonomy that adolescents may have in terms of treatment adherence, and the important influence that parents have in supporting them during the recovery period, dental provider discussions regarding pain management following third molar extractions ought to include both parties.

### Strengths and limitations

Strengths of the present study include that the interviews were conducted within a short time following the extraction, often just 2–3 weeks post-extraction, improving recall of the experience; the dataset including both adolescent and caregiver perspectives; the importance of the research topic; and the high level of qualitative research skill within our team. However, limitations must also be addressed. Because this was an exploratory qualitative study, our sample size was small. We decided upon our sample size before starting data collection, and it was assumed to be sufficient given the focused topic being examined and the relatively homogenous patient populations (e.g., ages 15–17 years of age, all having the same dental procedure), but thematic saturation may still have not been sufficiently reached. All study participants were recruited from a single health care system in the Midwest where a small number of oral surgeons performed all the extractions described and the populations is less racially and ethnically diverse than the national population. Additionally, several parents expressed having higher education or occupations in healthcare-related fields but the extent of parental education factors could not be explored because parental demographics were not collected or used in the sampling strategy. Findings may not generalize to other communities and populations, such as households without insurance coverage, or dentists. Also, we did not obtain information regarding the number of opioids prescribed based on the provider.

### Conclusions

Our study sheds light on pain management decision-making for youth undergoing third molar extractions and necessary information to convey to both adolescents and their parents/caregivers. The concordance in patient and parent perspectives identified throughout our data is encouraging from a public health perspective, and indicate the acceptance of non-opioid pain management strategies.

## Supplementary Information

**Additional file1: Appendix A.** Adolescent Interview Guide

**Additional file2: Appendix B. **Parent/Guardian Interview Guide

**Additional file3: Appendix C. **Code Book 

## Data Availability

The datasets generated and analyzed during the current study are not publicly available due their potentially identifiable nature but are available from the corresponding author on reasonable request.

## Abbreviation

REDCap: Research Electronic Data Capture
