# Supplementary material for: Pain management after third molar extractions in adolescents: a qualitative study
Source: BMC Pediatr. 2022 Apr 7;22:184. doi: 10.1186/s12887-022-03261-x (PMC8988337; doi:10.1186/s12887-022-03261-x)
Supplement: Supplementary file 1 — Additional file1: Appendix A. Adolescent Interview Guide [file 12887_2022_3261_MOESM1_ESM.pdf]

Appendix A. Adolescent Interview Guide

DIODE Supplement
Adolescent Interview Guide

Aim 1. To explore how adolescent patients and their parents make pain management decisions for dental extractions. Areas of exploration: We will examine and compare parent and adolescent knowledge, attitudes, and expectations regarding the use of opioids and non-opioids, as well as sources of influence regarding effective pain management for dental extractions.

All respondents

What was the reason for your most recent dental extraction?

Have you ever had a dental extraction before?
> If so, how did your most recent dental extraction compare to that other experience?

Have you ever had any other surgeries?
> If so, what was the surgery for? How did you manage post-op pain? Were you given medication for pain management (e.g., not antibiotics)? What medication were you prescribed?

Did the recent removal of your tooth/teeth go as planned or were there problems?
> If so, what kind of problems?

How did the dentist/oral surgeon advise you to manage the pain from your dental extraction(s)?
> Were you advised to use over-the-counter medications like Tylenol or ibuprofen?
> Were you given a prescription for pain medication?

What was the most important thing for you when deciding how to manage your pain?
> Immediate relief? Long-term pain management? Medication safety? Relieving pain and minimizing side-effects?

If NOT prescribed pain meds (i.e., told to use over-the-counter meds, ice, etc.)

What over-the-counter medications or other pain-relieving practices did you use following your dental extraction?
> How well did it work?
> Did you try anything else to manage the pain?
>> If so, what else did you try? How did you decide to try this? How much did it help?

Did your peers tell you about their experiences with similar extractions?
> If so, what did they tell you? How did that influence the way you through about managing your own pain?

Did your parents tell you about their experiences with similar extractions?
> If so, what did they say? How did that influence the way you thought about managing your own pain?

Did your talk with your parents prior to or following the extraction(s) about managing your pain?

How much input did you have regarding managing your pain?

If prescribed pain meds (i.e., opioids)

What medication were you prescribed?
What did you know about/what were you told about that medication?

Did your peers tell you about their experiences with similar extractions or taking that medication?

> If so, what did they tell you? How did that influence the way you through about managing your own pain?

Did you parents tell you about their experiences with similar extractions or taking that medication?

> If so, what did they tell you? How did that influence the way you through about managing your own pain?

What did the dentist/oral surgeon tell you about that medication?

Did you or your parents ask questions about that medication? If so, what questions were asked?

What do you think about the pain medication you were prescribed?

> How safe is it? How addictive is it? How effective is it compared with other pain medications, like Advil or Tylenol?

Was the prescription filled by you or one of your family members?

> If not, why not?
>
> > Did you discuss that decision with your parents or did they make that decision without you?
>
> If filled, did you take the medication?

Did you take it as prescribed?

> Did you take the amount of pills advised? Did you follow the recommended amount of time between doses?
>
> If not, why not?

Did it relieve your pain when taken as recommended? Was more effective than you expected? Did you have side effects?

Did you discuss the decision to take the medication in a non-prescribed manner with your parents?

Did you self-medicate or did your parents give you your medication?

How much input did you have regarding managing your pain?
