# Supplementary material for: Pain management after third molar extractions in adolescents: a qualitative study
Source: BMC Pediatr. 2022 Apr 7;22:184. doi: 10.1186/s12887-022-03261-x (PMC8988337; doi:10.1186/s12887-022-03261-x)
Supplement: Supplementary file 2 — Additional file2: Appendix B. Parent/Guardian Interview Guide [file 12887_2022_3261_MOESM2_ESM.pdf]

Appendix B. Parent/Guardian Interview Guide

DIODE Supplement
Parent/Guardian Interview Guide

Aim 1. To explore how adolescent patients and their parents make pain management decisions for dental extractions. Areas of exploration: We will examine and compare parent and adolescent knowledge, attitudes, and expectations regarding the use of opioids and non-opioids, as well as sources of influence regarding effective pain management for dental extractions.

All respondents

What was the reason for your child's most recent dental extraction?

Have you or your child ever had a dental extraction before?

Have you or your child ever had any other surgeries?

> If so, what was the surgery for? How was post-op pain managed? Were you or your child given medication for pain management (e.g., not antibiotics)? What medications were prescribed?

Did the recent removal of your child's the tooth/teeth go as planned or were there problems?

> If so, what kind of problems?

How did the dentist/oral surgeon advise you and/or your child to manage the post-op pain?

> Were you or your child advised to use over-the-counter medications like Tylenol or ibuprofen?
> Was your child given a prescription for pain medication?

What was the most important thing for you when deciding how to manage your child's pain?

> Immediate relief? Long-term pain management? Medication safety? Relieving pain and minimizing side-effects?

If NOT prescribed pain meds (i.e., told to use over-the-counter meds, ice, etc.)

What over-the-counter medications or other pain-relieving practices did you use following your child's most recent dental extraction?

> How well did it work?
> Did you try anything else to manage your child's pain?
>> If so, what else did you try? How did you decide to try this? How much did it help?

Did you talk with your child about your own personal experiences with similar extractions?

> If so, what did you say?

Did you talk with your child prior to or following the extraction(s) about the plan to manage their pain?

How much input did your child have regarding managing their pain?

If prescribed pain meds (i.e., opioids)

What medication was prescribed?

What did you know about/what were you told about that medication?

Did you talk with your child about your own experiences taking that medication or similar medications?

If so, what did you tell them?

What did the dentist/oral surgeon tell you and/or your child about that medication?

Did you or your child ask questions about that medication? If so, what questions were asked?

What do you think about that medication?

How safe is it? How addictive is it? How effective is it compared with other pain medications, like Advil or Tylenol?

Was the prescription filled by you or one of your family members?

If not, why not?

Did you discuss that decision with your child or did you make that decision independently?

If filled, did your child take the medication?

Did your child take it as prescribed?

Did your child take the amount of pills advised? Did you follow the recommended amount of time between doses?

If not, why not?

Did it relieve your child's pain when taken as recommended? Was more effective than you expected?
Did your child have side effects?

Did you discuss the decision to take the medication in a non-prescribed manner with your children?

Did your child self-medicate or did your give the medication to your child?

How much input did your child have regarding managing their pain?
