# Supplementary material for: Pain management after third molar extractions in adolescents: a qualitative study
Source: BMC Pediatr. 2022 Apr 7;22:184. doi: 10.1186/s12887-022-03261-x (PMC8988337; doi:10.1186/s12887-022-03261-x)
Supplement: Supplementary file 3 — Additional file3: Appendix C. Code Book [file 12887_2022_3261_MOESM3_ESM.pdf]

Appendix C. Code Book

Project: DIODE Supplement_Dyads (Codes and Definitions)

- **Complex extraction**

  difficulties during extraction
- ***Dentist-Oral Surgeon info***

  what were they told about pain management by the dentist/oral surgeon
- ***Other pain mgmt strategies***

  Non-medicine pain management strategies (e.g., ice)
- ***Pain meds-OTC***

  non-opioid medications recommended
- ***Pain meds-Rx***

  opioids prescribed
- ***Pain mgmt goal***

  most important thing when deciding how to manage pain
- ***Pain mgmt-adolescent***

  pain management actvities performed by the adolescent
- ***Pain mgmt-parent***

  pain management activities performed by the parent
- ***Parent input***

  pain management advice from parent
- ***Peer input***

  pain management advice from peers
- ***Prior surgeries***

  prior surgery experiences
- ***Questions asked***

  questions asked of dentist by parent or adolescent
- ***Rx attitudes***

  what did you think about the pain meds prescribed
- ***Rx filled***

  was the prescription filled
- ***Rx taken as prescribed***

  was the prescription taken as prescribed
- ***Shared decision making***

  shared decision making between parent and adolescent
